# Increased prevalence of non-communicable physical health conditions among autistic adults

**DOI:** 10.1177/1362361320953652

**Published:** 2020-09-09

**Authors:** Elizabeth Weir, Carrie Allison, Varun Warrier, Simon Baron-Cohen

**Affiliations:** University of Cambridge, UK

**Keywords:** adults, autism spectrum disorders, health services, medical comorbidity

## Abstract

Autistic individuals may be at risk of premature mortality, and physical health comorbidity increases this risk; however, most studies fail to include older autistic adults or consider lifestyle-related factors that affect health. We developed an anonymous, online physical health survey. The final sample included n = 2368 individuals (mean age = 41.42), and of these, n = 1156 were autistic individuals (mean age = 40.98). We utilized three sex-stratified statistical models to determine the prevalence of cancer, cardiovascular conditions, respiratory conditions, and diabetes. All three models indicate that autistic females are more likely to have cardiovascular conditions, respiratory conditions, asthma, low blood pressure, arrhythmias, and prediabetes than non-autistic females, and autistic males are more likely to have arrhythmias than non-autistic males; these results suggest that autistic individuals carry increased risks for these conditions when compared to the general population, even after controlling for age, ethnicity, education level, body mass index, smoking, and alcohol use. Further, these risks may differ depending on biological sex for autistic individuals. Autistic adults, and particularly autistic females, have greater and wider-ranging risks than previously thought, even after controlling for demographic and lifestyle-related factors. Although this is a large sample of autistic adults across the lifespan, future research should employ larger, population-based samples to confirm these findings.

**Lay abstract:**

Previous research indicates autistic individuals die at a younger age than others and that this is possibly due in part to chronic physical health conditions. The present study used an anonymous, online survey to determine how common certain physical health conditions are among autistic adults, compared with non-autistic adults. We found autistic adults are more likely to develop heart conditions, lung conditions, and diabetes than non-autistic adults. Autistic females may be at higher risk of developing certain conditions (including respiratory conditions, asthma, and prediabetes) than autistic males. Finally, autistic individuals have increased health risks even when considering lifestyle factors (such as smoking, alcohol, and body mass index). This is still a relatively small study, and future research needs to confirm these findings and identify why these risks exist.

## Introduction

Autism spectrum conditions (henceforth autism) are lifelong, neurodevelopmental conditions characterized by social and communication difficulties; markedly restricted, repetitive interests and behavior; and differences in cognitive profile, including atypical sensory perception and information processing, motor abilities, and intellectual ability (American Psychiatric Association, 2013). Although autism was historically classified as a rare condition, prevalence estimates have increased in recent years, and now approximately 1%–2% of the population are diagnosed as autistic (likely due to expansion of diagnostic criteria and greater recognition of the condition due to increased awareness; Baio, 2018). In addition, there is a sex bias in autism, with males being diagnosed approximately 3 to 4 times more frequently than females (Baio, 2018; Loomes et al., 2017). Due to historic increases in prevalence, greater numbers of autistic individuals continue to reach adolescence and adulthood.

Autistic individuals are at higher risk of premature mortality than non-autistic individuals, with some evidence of further increased risks for those with intellectual disability and for autistic females; in addition, chronic physical health conditions or poor physical health are associated with premature mortality for autistic individuals across the spectrum of intellectual ability (Bishop-Fitzpatrick et al., 2018; DaWalt et al., 2019; Hirvikoski et al., 2016; Hwang et al., 2019; Woolfenden et al., 2012). Despite these risks, there are very few studies of autistic adults with a large enough sample size to accurately identify medical conditions that frequently co-occur with autism.

To date, eight large-scale studies have attempted to identify the common medical comorbidities of autistic adults in a variety of ways. However, almost none of these studies effectively consider physical health across the lifespan: three studies only include autistic individuals younger than 40 years, and additional three each include less than 325 autistic people over the age of 40. Despite these limitations, all six of these studies indicate that autistic individuals are at increased risk of nearly every physical health condition asked about; taken collectively, this includes increased risk of neurological conditions and central nervous system (CNS) anomalies (and, in particular, epilepsy/seizure disorders), cardiovascular conditions (including dyslipidemia/lipid metabolism disorders, hypertension, and stroke), gastrointestinal conditions, metabolic conditions and diabetes (including Type I diabetes, being overweight, and obesity), pulmonary/respiratory conditions (including asthma and chronic obstructive pulmonary disease (COPD)), immune/autoimmune conditions, endocrine conditions (including thyroid conditions), pubertal disorders, genitourinary/kidney conditions, musculoskeletal conditions, sleep disturbances/disorders, nutritional conditions, muscular dystrophy, genetic disorders, physical disabilities, skin disorders, infections, hematological disorders, jaw and teeth disorders, ENT (ear, nose, and throat) conditions, and ophthalmological conditions for young autistic individuals compared to young non-autistic individuals (Croen et al., 2015; Davignon et al., 2018; Fortuna et al., 2016; Kohane et al., 2012; Vohra et al., 2017; Weiss et al., 2018).

It should be noted that some of these studies reported that autistic individuals were at relatively lower risk for some conditions compared to non-autistic individuals, including migraines, musculoskeletal conditions, genitourinary conditions, cancer, cardiovascular disorders, diabetes, and respiratory conditions (Croen et al., 2015; Davignon et al., 2018; Fortuna et al., 2016; Vohra et al., 2017); however, there are conflicting results across different studies for each of these conditions. For example, there is genetic and epidemiological evidence to suggest that autistic individuals have a different likelihood of developing cancer than those in the general population; yet, due to methodological differences across studies (primarily regarding limited sample sizes and age range), the direction of this risk is still under debate (Chiang et al., 2015; Crawley et al., 2016; Crespi, 2011; Darbro et al., 2016; Kao et al., 2010; Mouridsen et al., 2016; Vohra et al., 2017; Wen et al., 2016). Even taking these limitations into account, there appears to be a pattern of increased health burden for most physical health conditions among autistic individuals.

Further, three studies (including two of the above) of primarily younger autistic adults considered healthcare utilization, and showed that autistic individuals are more likely than non-autistic individuals to have outpatient, inpatient, primary care, emergency room, mental health/psychiatric, neurology, speech therapy, and laboratory visits; have prescription drug claims; and be hospitalized (Vohra et al., 2017; Weiss et al., 2018; Zerbo et al., 2018). Thus, it is unsurprising that they also had higher mean annual expenditure for outpatient, primary care, emergency, mental health/psychiatry, neurology, home health care, and skilled nursing visits; prescription drug claims; and overall healthcare than non-autistic individuals (and the presence of a psychiatric or physical health comorbidity increased expenditure further; Vohra et al., 2017; Weiss et al., 2018; Zerbo et al., 2018).

In addition to the six studies of primarily younger adults, two recent studies attempted to quantify increased physical health comorbidity burden for autistic adults across the lifespan. The first utilized census records in the Scottish population (n = 6649 autistic adults) and identified increased risks for autistic adults over the age of 25 years compared to non-autistic adults in all the categories surveyed, with odds ratios (ORs) of 6.2 and 2.6 for physical disability and other conditions (which may include physical health conditions), respectively (Rydzewska et al., 2018). Unfortunately, the census data did not provide any information about physical health specifically, or about any particular physical health conditions.

The second utilized a large, cross-sectional sample of Medicare data (n = 4685 autistic adults) to determine whether older autistic adults (specifically those aged 65 years or older) are at increased risk of physical and mental health conditions (Hand et al., 2020). They found that, compared to individuals in the general population, autistic adults were at increased risk of nearly every condition tested (except menopausal disorders, multiple sclerosis, back conditions, and substance use disorders, whose findings were non-significant); for physical health specifically, the study confirmed previous findings from studies of younger adults (increased risk of all other metabolic, neurological, respiratory, gastrointestinal, circulatory/cardiovascular, and musculoskeletal conditions) and identified that older autistic adults were also at increased risk of cancer, heart disease, and cerebrovascular disease (Hand et al., 2020). The findings of this study highlight the need for research in autistic adults across the lifespan (and particularly in older adults), as they may have greater and/or different risks than younger autistic individuals.

Finally, in addition to increased risk of specific health conditions, some studies indicate that there may be risk factors that have knock-on effects for autistic adults, placing them at even higher likelihood of developing a variety of physical health conditions than those in the general population. First, all the studies that investigated potential sex differences in healthcare burden found that autistic females had even greater risks for most physical health conditions and lower reported health status overall than autistic males (Croen et al., 2015; Davignon et al., 2018; Fortuna et al., 2016; Rydzewska et al., 2018); these findings are in line with mortality data which show that autistic females are at uniquely increased risk of premature mortality even compared to autistic males (Hirvikoski et al., 2016; Hwang et al., 2019; Woolfenden et al., 2012). Based on these findings, new research should focus on identifying physical health risks in autistic males and females separately, as well as quantifying relative risk between these groups. Second, two recent studies show evidence of cardiovascular risk factors that may contribute to greater risk of a variety of conditions among autistic individuals compared to non-autistic individuals, including reduced cardiorespiratory capacity and reduced heart rate variability (Bricout et al., 2018; Thapa et al., 2019). Third, a systematic review and meta-analysis confirms the findings of Croen et al. and Hand et al. to suggest that autistic individuals are at greater risk of obesity than non-autistic people (Croen et al., 2015; Hand et al., 2020; Zheng et al., 2017). Obesity has been shown to be significantly associated with increased risk of several non-communicable diseases, including Type II diabetes, cancer, cardiovascular conditions, and asthma (Guh et al., 2009). Fourth, there is still significant debate regarding frequency of substance use and abuse among autistic adults, and how this may affect physical health of autistic adults. Several small studies indicate decreased substance use among autistic individuals overall (Croen et al., 2015; Davignon et al., 2018; Fortuna et al., 2016; Vohra et al., 2017). Conversely, other studies have suggested increased substance use problems among autistic adults, including a large population-based study in Sweden (n = 26,986 autistic individuals) which suggests that autism is a risk factor for substance use–related problems, with elevated risks even for relatives of autistic individuals (Butwicka et al., 2017; Weiss et al., 2018). Substance use/abuse increases risks of respiratory problems (including asthma), cancer, heart disease, hypertension, heart attack, stroke, reproductive morbidity, diabetes, liver damage/disease, and sleep conditions (Schulte & Hser, 2014). We are unaware of any large-scale studies of physical health comorbidity burden of autistic adults that take into account lifestyle factors like obesity, smoking, or alcohol use. Thus, it appears that autistic adults, and particularly autistic females, may experience higher rates of nearly all types of physical health conditions, which may affect both quality and length of life.

Considering the significantly increased risks of premature mortality among autistic adults, it is of particular import to identify which physical health conditions are contributing the most risk. In 2016, the World Health Organization (WHO) reported that 71% of worldwide mortality was accounted for by four non-communicable diseases: cancers, cardiovascular conditions, respiratory conditions, and diabetes; therefore, research on the physical health of autistic adults should focus on identifying differences in these key areas to maximize potential public health impact. To create effective interventions and reduce risk of physical health conditions and premature mortality, new research must aim to identify risks for cancers, cardiovascular conditions, respiratory conditions, and diabetes across the lifespan; quantify risks for autistic females specifically; and determine whether lifestyle factors may serve as key points of intervention for reducing risk.

## Methods

### The survey

We developed an anonymous, online physical health survey that included questions about demographic information, a short version of the Autism Spectrum Quotient (a measure of autistic traits, AQ-10; Allison et al., 2012; Greenberg et al., 2018), daily habits (including exercise, diet, sleep, disability, and social/sexual history), as well as personal and family medical histories of common medical conditions. The medical history sections included lists of conditions from the broad categories of cancer; cardiovascular; respiratory; gastrointestinal; hormonal/reproductive; musculoskeletal; neurological; eye; ear, nose, and throat; liver and kidney; blood and lymph; skin; diabetic; and autoimmune conditions.

The questionnaire comprised 512 questions, asking about more than 150 medical conditions and daily habits. To avoid survey fatigue, the medical history sections utilized a tiered structure. Participants were directed to first select the broad health category that corresponded to each of their health conditions and, based on their selections, additional questions appeared with lists of common conditions within the selected category. In addition, each category list included a free text box where participants could report a diagnosis of any condition about which the survey did not specifically inquire. Further, there was a final text box at the end of the medical history section asking if there was any additional information that participants wished to provide about their health or medical history. As the survey used a tiered design, we scanned all free text boxes to ensure that conditions listed throughout the personal medical history section were appropriately coded and that conditions listed met our requirements for inclusion. Medical conditions were ascertained by asking “Which of the following conditions have you ever had? Please select all that apply:” for each of the selected categories, indicating that individuals should provide a cumulative medical history across their lifespan rather than over a specified time limit (e.g. the last 5 years). See Supplemental Information for additional details. The conditions were selected using online, publicly available materials from the National Health Service (NHS), Cancer Research United Kingdom (CRUK), National Institute for Health and Care Excellence (NICE), National Institutes of Health (NIH), and the WHO.

### Recruitment

This study utilized a convenience sampling framework, recruiting online participants via the Cambridge Autism Research Database (CARD), Autistica’s Discover Network, autism support groups and charities (including the Autism Research Trust), and social media (specifically Twitter and Facebook). These sampling methods may be biased toward those with autism or an interest in autism, as we advertised to groups/forums related to autism; however, all advertisements encouraged participation from both autistic and non-autistic individuals. In addition, we used Facebook to advertise our study to the general population, in an attempt to limit bias from recruiting individuals via only autism-specific groups/forums; in this phase of recruitment, both autistic and non-autistic Facebook users from around the world were invited to participate. The study aimed to include an international cohort of individuals from all countries, and respondents from over 60 different countries were included in the sample.

Survey collection took place between February 2018 and August 2019. There were two periods within this time where survey collection was paused; no changes were made to the survey during these periods. We performed a sensitivity analysis covarying for time period in Model 2 and used z tests to determine if the pauses in survey collection affected our results; we found no statistically significant differences in the results.

### The cohort

N = 3657 individuals accessed the survey. Participants included any individual who was aged at least 16 years and consented to participate. We excluded 1102 individuals due to “incomplete” response, meaning that they exited the survey before providing their medical history. Nine hundred fourteen of the individuals excluded due to incomplete response (83%) did not even complete the demographics section of the survey (and answered no questions related to lifestyle or physical health), making their responses unusable for this analysis. Some questions were optional, and individuals were not excluded from analysis if they chose to skip optional questions; however, all questions related to medical history were required. We excluded one individual who indicated “Other” for their biological sex, as our analysis strategy splits participants by biological sex.

As the survey was anonymous, we used an algorithm to exclude potential duplicate responses (n = 108). We excluded all records that matched a previous record on 11 criteria (autism diagnosis (yes/no), specific autism diagnosis, type of diagnosing practitioner, year of autism diagnosis, country of residence, biological sex, current gender identity, education level, age, maternal age at birth, and paternal age at birth).

We used a case–control design to divide our sample into an autistic cohort and a control cohort. Our autistic cohort included individuals with an autism diagnosis, provided by a medical practitioner. Autism diagnoses were self-reported; however, we asked participants to provide additional information to verify their diagnosis, such as the type of practitioner who diagnosed them (e.g. psychiatrist, clinical psychologist, and pediatrician), year of their diagnosis, specific diagnosis (e.g. autism spectrum disorder, Asperger’s, etc), and whether they have a syndromic form of autism. As we followed a case–control design, individuals who self-diagnosed as autistic, suspected autism, or were waiting to be assessed for autism were excluded from both the autistic and non-autistic (control) groups (n = 44). We also excluded those who reported a syndromic form of autism (n = 34) that carries known physical health risks (i.e. Klinefelter/XXY and PTEN hamartoma tumor syndrome). Our control population included any individuals who do not have autism or suspect autism; as noted above, individuals who report a self-diagnosis of autism, suspected autism, or who are waiting to be assessed with autism were excluded from the control group to preserve the case–control design. There were no additional exclusion criteria for controls. The final sample comprised n = 2368 individuals, including 1156 autistic individuals.

### Analysis

As noted above, approximately 71% of worldwide mortality can be attributed to four non-communicable diseases: cancers, cardiovascular conditions, respiratory conditions, and diabetes. Considering the increased risks of premature mortality to autistic individuals, we limited our analyses to these four broad categories of physical health and tested risks overall, as well as by condition for all conditions reported with at least 1% prevalence in the cohort being tested. By restricting our analyses to these conditions that account for such a large proportion of worldwide mortality, we were able to employ more in-depth analyses on the differences in prevalence between autistic and non-autistic adults, as well as consider effects of lifestyle factors (smoking, alcohol, body mass index (BMI)), age, and sex. As such, we designed this analysis with the hopes of identifying specific points of intervention for healthcare providers, autistic individuals, and caregivers which, in turn, might serve to reduce risks of chronic disease and premature mortality among autistic individuals.

We used *R Version 3.6.2* to employ three sex-stratified statistical models, comparing rates of medical conditions among autistic females versus non-autistic females, and separately, autistic males versus non-autistic males. We chose to segregate all our analyses by biological sex, as mortality and prevalence of physical health conditions in the general population vary greatly by biological sex (Oksuzyan et al., 2008; Verbrugge et al., 1987), and some studies suggest that autistic females may be at even higher risk of health conditions and premature mortality (Croen et al., 2015; Davignon et al., 2018; Hirvikoski et al., 2016; Hwang et al., 2019; Rydzewska et al., 2018; Woolfenden et al., 2012).

The first model used sex-stratified one-tailed Fisher’s exact tests. The second utilized sex-stratified binomial logistic regression (specifically Firth’s Bias-Reduced Logistic Regression using the R package “logistf”) and controlled for demographic factors, including age, ethnicity, country of residence, and educational level (as a crude measure of socioeconomic status). The third also utilized sex-stratified binomial logistic regression and controlled for the same demographic factors, as well as some factors that may be related to lifestyle choices and daily habits, including BMI, alcohol use, and smoking. We used frequency of current alcohol consumption, as measured by the number of days per week with the following options: “I do not consume alcoholic beverages,” “1–2 days per week,” “3–4 days per week,” and “5–7 days per week,” to quantify alcohol use among participants. We used highest frequency of smoking ever, as measured by the regularity of smoking when smoking most frequently with the following options: “I have never smoked regularly,” “Monthly,” “Weekly,” and “Daily,” to quantify smoking among participants. We employed one imputation (using predictive mean matching within the Multivariate Imputation via Chained Equations (MICE) package) to address missingness in the data for the variables of age, BMI, educational level, ethnicity, country of residence, smoking, and alcohol use (Azur et al., 2011). We used imputation exclusively for the covariates; therefore, the imputation only affected Model 2 and Model 3. We employed the false discovery rate correction to minimize the risks of Type I errors from multiple testing and used a p threshold of 0.05 for all three models (Benjamini & Hochberg, 1995). We also conducted z tests to compare the relative risk of conditions between females and males, to determine whether our study replicates previous findings and suggests that there is relatively larger risk of physical health conditions among autistic females in our sample, compared to autistic males. Finally, we performed post hoc analyses on Model 2 to investigate the interaction of age and diagnosis on the likelihood of developing each of our outcomes of interest.

### Ethical approval

This study received ethical approval (HBREC.2017.28) from the University of Cambridge Human Biology Research Ethics committee.

## Results

The mean age of the autistic group was 40.98 years (SD = 14.41) and the mean age of the control group was 41.84 years (SD = 15.50). The groups did not differ in age (χ^2^ = 81.878, df = 67, p > 0.05). Our sample was biased toward females, White individuals, and UK residents, and there were significant group differences. This was expected based on the methodology and recruitment strategies employed. Table 1 includes a summary of demographic information for both the autistic and non-autistic participants.

**Table 1.** Participant demographics.

Characteristics	Autism (n = 1156)	Controls (n = 1212)	p values (significance level)
Age (years), mean (SD)	40.98 (14.41)	41.84 (15.50)	0.304
Age (years), categories, N (%)			
16–29	298 (25.78)	311 (25.66)	
30–39	246 (21.28)	245 (20.21)	
40–49	243 (21.02)	256 (21.12)	
50–59	212 (18.34)	210 (17.33)	
60–69	108 (9.34)	124 (10.23)	
70+	25 (2.16)	52 (4.29)	
Missing	24 (2.08)	14 (1.16)	
Biological sex, N (%)			0.017 (*)
Female	738 (63.84)	830 (68.48)	
Male	418 (36.16)	382 (31.52)	
Missing	0	0	
Ethnicity, N (%)			5.074 × 10^−7^ (***)
White	1021 (88.32)	1029 (84.90)	
Mixed race	74 (6.40)	75 (6.19)	
Asian	18 (1.56)	41 (3.38)	
Latin American/Hispanic	7 (0.61)	23 (1.90)	
Arab/Middle Eastern	0 (0.00)	17 (1.40)	
Jewish	16 (1.38)	17 (1.40)	
African/Black/Caribbean	6 (0.52)	9 (0.74)	
Missing	14 (1.21)	1 (0.08)	
Education, N (%)			1.609 × 10^−16^ (***)
No formal qualifications	53 (4.58)	13 (1.07)	
Further vocational qualifications	206 (17.82)	140 (11.55)	
Secondary school/high school	208 (17.99)	171 (14.11)	
University undergraduate	348 (30.10)	358 (29.54)	
University postgraduate	339 (29.33)	527 (43.48)	
Missing	2 (0.17)	3 (0.25)	
Country of residence			3.126 × 10^−7^ (***)
United Kingdom	821 (71.02)	765 (63.12)	
United States	118 (10.21)	175 (14.44)	
Germany	30 (2.60)	33 (2.72)	
Australia	32 (2.77)	21 (1.73)	
Canada	25 (2.16)	24 (1.98)	
Ireland	13 (1.12)	30 (2.48)	
The Netherlands	28 (2.42)	8 (0.66)	
Other	89 (7.70)	156 (12.87)	
Missing	1 (0.09)	3 (0.25)	
Body mass index (kg m^−2^), mean (SD)	27.74 (8.30)	26.60 (6.87)	3.999 × 10^−3^ (***)
Missing	25 (2.16)	20 (1.65)	
Most frequent smoking, N (%)			0.031 (*)
Never	783 (67.73)	779 (64.27)	
Monthly	2 (0.17)	9 (0.74)	
Weekly	25 (2.16)	41 (3.38)	
Daily	346 (29.93)	382 (31.52)	
Missing	0	1 (0.08)	
Current alcohol frequency (days per week), N (%)			2.344 × 10^−20^ (***)
0	675 (58.39)	469 (38.70)	
1–2	292 (25.26)	475 (39.19)	
3–5	119 (10.29)	183 (15.10)	
6–7	69 (5.97)	84 (6.93)	
Missing	1 (0.09)	1 (0.08)	

These are demographic data before imputation. The results remain highly similar after imputation. p values were from Pearson’s chi-square test (categorical) or from a Mann–Whitney U test (means).

* p<0.05. **p<0.01. ***p<0.001

**Figure 1.**
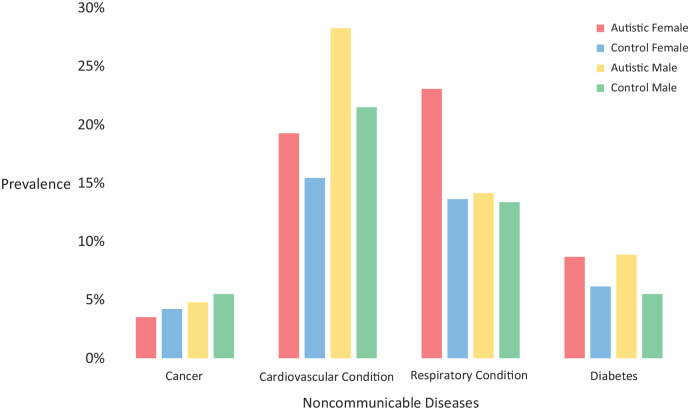
Unadjusted prevalence of cancers, cardiovascular conditions, respiratory conditions, and diabetic conditions.

**Figure 2.**
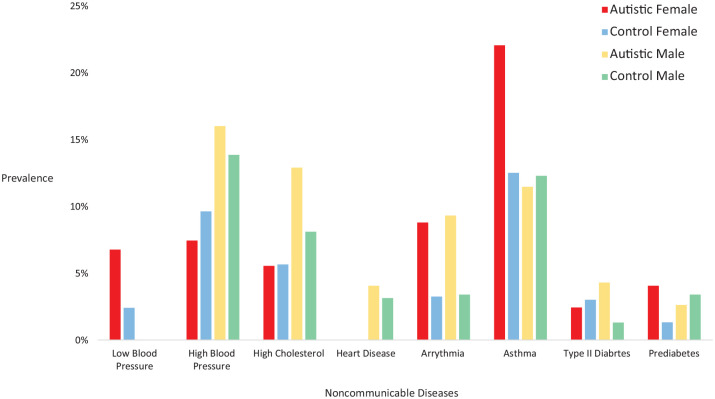
Unadjusted prevalence of specific non-communicable diseases.

The following figures illustrate the reported prevalence of non-communicable diseases among autistic and non-autistic adults. Figure 1 shows the prevalence of the four large categories of non-communicable diseases that cumulatively account for the majority of worldwide mortality each year, as reported by the WHO. Figure 2 clarifies the prevalence of the specific conditions with at least 1% prevalence among the cohort being tested.

### Summary of results from all three models

Using one-tailed Fisher’s exact tests, Model 1 provided us with an unadjusted model and is, thereby, the least conservative in estimating group differences between autistic and non-autistic adults. Model 1 found that autistic females are at increased risk of cardiovascular conditions overall and respiratory conditions overall, as well as specifically for low blood pressure, arrhythmias, asthma, and prediabetes compared to non-autistic females. Autistic males have elevated risks of cardiovascular conditions overall, as well as high cholesterol, arrhythmias, and Type II diabetes, compared to non-autistic males. Supplemental Table 1 provides the complete results for Model 1.

Model 2 employed binomial logistic regression and controlled for demographic factors to reduce bias from our sampling methods (age, ethnicity, country of residence, and education-level). Model 2 found that autistic females have higher rates of cardiovascular, respiratory, and diabetic conditions overall, as well as specifically for low blood pressure, arrhythmias, asthma, and prediabetes than non-autistic females. Compared to non-autistic males, autistic males are at increased risk of arrhythmias. Supplemental Table 2 provides the complete results for Model 2.

Finally, Model 3 also used binomial logistic regression and controlled for the same demographic factors and also controlled for factors that could be related to lifestyle and daily habits, including BMI, alcohol use, and smoking. Thus, Model 3 attempts to quantify risks between autistic and non-autistic females and males, respectively. Model 3 found that autistic females are more likely than non-autistic females to have cardiovascular, respiratory, and diabetic conditions overall, as well as specifically for low blood pressure, arrhythmias, asthma, and prediabetes; in addition, it found higher risk of arrhythmias for autistic males than for non-autistic males. Supplemental Table 3 provides the complete results for Model 3. Table 2 shows the results of the three models together for each of the categories or conditions tested.

**Table 2.** Summary of all three sex-stratified models.

Conditions	Model 1			Model 2			Model 3			Sig. models
	OR	95% CI	Sig.	OR	95% CI	Sig.	OR	95% CI	Sig.	
Cancer										
Female	0.830	0.517, Inf		1.098	0.627, 1.911		1.068	0.605, 1.872		
Male	0.864	0.484, Inf		0.840	0.431, 1.628		0.739	0.367, 1.473		
Cardiovascular										
Female	1.306	1.039, Inf	*	1.511	1.139, 2.010	**	1.383	1.033, 1.854	*	1, 2, 3
Male	1.438	1.082, Inf	*	1.590	1.104, 2.302	†	1.542	1.056, 2.262		1
Respiratory										
Female	1.898	1.511, Inf	***	2.016	1.541, 2.647	***	2.054	1.557, 2.719	***	1, 2, 3
Male	1.067	0.745, Inf		0.955	0.623, 1.464		0.903	0.583, 1.399		
Diabetic										
Female	1.450	1.034, Inf	†	1.835	1.223, 2.771	**	1.692	1.109, 2.599	*	2, 3
Male	1.668	1.015, Inf	†	1.731	0.979, 3.132		1.509	0.822, 2.830		
Low blood pressure										
Female	2.941	1.842, Inf	***	2.900	1.716, 5.073	***	2.541	1.494, 4.467	**	1, 2, 3
High blood pressure										
Female	0.755	0.549, Inf		0.965	0.650, 1.426		0.873	0.578, 1.313		
Male	1.185	0.838, Inf		1.292	0.850, 1.975		1.158	0.743, 1.812		
High cholesterol										
Female	0.980	0.666, Inf		1.435	0.892, 2.313		1.382	0.850, 2.248		
Male	1.679	1.110, Inf	*	1.523	0.924, 2.542		1.451	0.867, 2.456		1
Heart disease										
Male	1.307	0.652, Inf		1.146	0.520, 2.593		1.121	0.501, 2.562		
Arrhythmia										
Female	2.871	1.913, Inf	***	2.941	1.854, 4.783	***	2.928	1.829, 4.804	***	1, 2, 3
Male	2.917	1.644, Inf	**	2.979	1.556, 6.047	*	3.085	1.561, 6.458	*	1, 2, 3
Asthma										
Female	1.978	1.565, Inf	***	2.069	1.571, 2.736	***	2.092	1.576, 2.789	***	1, 2, 3
Male	0.925	0.631, Inf		0.832	0.527, 1.313		0.796	0.499, 1.267		
Type II diabetes										
Female	0.805	0.455, Inf		1.237	0.637, 2.386		1.028	0.510, 2.055		
Male	3.388	1.374, Inf	*	2.483	0.995, 7.150		1.799	0.688, 5.266		1
Prediabetes										
Female	3.153	1.687, Inf	**	4.337	2.147, 9.395	***	4.379	2.102, 9.772	***	1, 2, 3
Male	0.767	0.353, Inf		1.075	0.461, 2.483		1.020	0.419, 2.474		

CI: confidence interval; OR: odds ratio; Sig.: significance level; Sig. models: significant models.

† p < 0.10. *p < 0.05. **p < 0.01. ***p < 0.001.

In addition, we performed an interaction analysis, again using binomial logistic regression, to determine if there was a significant interaction between age and autism diagnosis. There were marginally significant interactions between age and diagnosis for cardiovascular conditions overall for autistic females (compared to non-autistic females), as well as for high cholesterol and heart disease for autistic males (compared to non-autistic males); however, these results did not survive correction. Full results for this analysis are provided in Supplemental Table 4.

Finally, we performed z tests on Models 2 and 3 to determine if there was relatively increased risk for any of the physical health conditions tested, based on biological sex. Our results support that autistic females are significantly more likely than autistic males to have respiratory conditions overall, as well as asthma and prediabetes specifically. There were no significant differences between autistic males and females for any other condition tested; full results are provided in Table 3 above.

**Table 3.** Risks of conditions for autistic females versus autistic males.

Conditions	Model 2				Model 3			
	Female OR	Male OR	FDR p value	Significance level	Female OR	Male OR	FDR p value	Significance level
Cancer	1.098	0.840	0.876		1.068	0.739	0.655	
Cardiovascular	1.511	1.590	0.966		1.383	1.542	0.900	
Respiratory	2.016	0.955	0.018	*	2.054	0.903	8.754 × 10^−3^	**
Diabetic	1.835	1.731	0.966		1.692	1.509	0.900	
High blood pressure	0.965	1.292	0.630		0.873	1.158	0.655	
High cholesterol	1.435	1.523	0.966		1.382	1.451	0.900	
Arrhythmia	2.941	2.979	0.975		2.928	3.085	0.900	
Asthma	2.069	0.832	7.500 × 10^−3^	**	2.092	0.796	4.481 × 10^−3^	**
Type II diabetes	1.237	2.483	0.497		1.028	1.799	0.655	
Prediabetes	4.337	1.075	0.027	*	4.379	1.020	0.024	*

FDR: false discovery rate; OR: odds ratio.

We could not test sex differences for low blood pressure or heart disease, as these were only tested in one sex each (females and males, respectively) for the purposes of this study.

* p<0.05. **p<0.01.

## Discussion

By utilizing three statistical models, we were able to parse out risks to autistic adults more specifically than in previous studies. Results that demonstrate differences in physical health comorbidity across all three models suggest that these differences exist, regardless of both demographic and lifestyle-related factors. We found that, compared to non-autistic females, autistic females are more likely to have a cardiovascular condition (OR: 1.51), approximately twice as likely to have a respiratory condition (OR: 2.02) and asthma specifically (OR: 2.07), nearly 3 times as likely to have low blood pressure (OR: 2.90) and arrhythmias (OR: 2.94), and over 4 times as likely to have prediabetes (OR: 4.34) and that autistic males are also nearly 3 times more likely to have arrhythmias (OR: 2.98) than non-autistic males. It seems that both autistic females and males carry these increased risks, even when accounting for age, ethnicity, education level, country of residence, BMI, smoking, and alcohol use. Therefore, this suggests that autistic adults have these increased health risks, even after taking into account the potential risks associated with obesity and substance use problems (which may be higher in autistic individuals; Butwicka et al., 2017; Croen et al., 2015; Weiss et al., 2018; Zheng et al., 2017).

There is also limited evidence of elevated risks of diabetic conditions (OR: 1.84) for autistic females, as well as greater risks of cardiovascular conditions (OR: 1.59), high cholesterol (OR: 1.52), and Type II diabetes (OR: 2.48) for autistic males. However, these results are only supported by one or two models, so they need to be taken as preliminary evidence at this stage.

In sum, the present results confirm previous findings of increased risks for diabetes (including prediabetes and Type II diabetes), cardiovascular conditions (including high cholesterol), and asthma (Croen et al., 2015; Davignon et al., 2018; Weiss et al., 2018). Our findings do not confirm the results reported by Vohra and colleagues (2017), which found decreased risk of cardiovascular, respiratory, and diabetic conditions; however, our participants may display greater physical health burden due to our sample’s wider age range, as the earlier study only included 255 autistic participants over the age of 40 years. Finally, our results extend previously reported conditions for autistic adults to include elevated risk of low blood pressure for autistic females, as well as increased risk of arrhythmias for both autistic females and autistic males.

We also tested the effects of age on developing these physical health conditions. Within our sample, we found no significant interaction between autism diagnosis and age. These results could reflect true patterns, suggesting that increasing age may similarly affect risk of developing chronic physical health conditions among both autistic and non-autistic individuals; alternatively, our sample could be underpowered to detect the interaction between autism diagnosis and age. Another important point to note is that we asked participants to report whether they have ever had a condition and did not ask the age at which the condition was diagnosed. As such, our study may not have the sensitivity to detect effects of age. This may be particularly true for conditions, such as asthma, which are frequently diagnosed during childhood; thus, our results reflect the cumulative likelihood of developing these conditions across the lifespan of each individual, rather than the conditions diagnosed exclusively during adulthood or over a particular time frame.

In regard to sex differences, our results suggest that autistic females may have excess risk of developing respiratory conditions, asthma, and prediabetes compared to autistic males. We found no significant differences in any other conditions tested, which again may reflect true differences, or we may be underpowered due to undersampling of males. Increased risks among autistic females reported in our study align with previous findings from both mortality data (Hirvikoski et al., 2016; Woolfenden et al., 2012) and physical health studies (Croen et al., 2015; Davignon et al., 2018; Rydzewska et al., 2018), but expand the specific list of conditions for which autistic females may have excess risk. Further, these findings may have important policy implications for healthcare providers of autistic females: this group may require additional healthcare surveillance and patient education to reduce risk of developing physical health comorbidities.

### Limitations

While both autistic males and females appear to have increased physical health risks, this study is underpowered to provide reliable effect size differences between autistic and non-autistic adults, and particularly for rare medical conditions. Due to its relatively small sample size for epidemiological research, it may be subject to the “winner’s curse,” resulting in artificially inflated ORs. Instead, it provides evidence of relatively increased risk for autistic adults across the lifespan, which should be investigated further. In addition, future research should attempt to recruit larger samples and to consider differences in risk of physical health comorbidity across age groups, by comparing prevalence of health conditions among young, middle-aged, and older adults separately.

This study used a self-report survey measure, rather than medical records. Thus, our study relied on participants to provide an accurate account of their physical health conditions, which may introduce bias into our sample. The study may also be subject to sampling bias, as we advertised the study via various sources of social media, and autism support groups and charities. Further, the survey was only available in English, and this may have biased our sample as well.

Another limitation is that our methodology excluded participants who are unable to participate in an online, self-report survey. This likely limited our analyses to individuals with access to the Internet, as well as average intellectual ability and physical functioning, which is not representative of the entire autistic population. Evidence from mortality data (Hirvikoski et al., 2016; Hwang et al., 2019; Woolfenden et al., 2012) suggests that autistic individuals with intellectual disability may be at particularly high risk for premature mortality and that the specific risk factors for premature mortality in this group may be different from autistic individuals without intellectual disability. Future research should focus on identifying the physical health risks of autistic individuals across the spectrum of intellectual ability and physical functioning.

Our control population may not be representative of the general population, as our recruiting methods may have been more likely to reach individuals with an interest in autism, or who may suspect that they are autistic. We excluded individuals from both the autistic and control groups who reported self-diagnosis of autism or suspected they are autistic. However, even with these exclusions, we cannot preclude the possibility that our control group may include some individuals with undiagnosed autism, high autistic traits, a broad autistic phenotype, or genetic liability for autism. Thus, our results may underestimate true group differences between autistic and non-autistic individuals.

It was particularly challenging to recruit males (both autistic and non-autistic) to the sample; this was expected, as self-report surveys are typically heavily biased toward female respondents, and this pattern is reported specifically for online surveys focused on healthcare (Aerny-Perreten et al., 2015; Cheung et al., 2017; Cull et al., 2005; Listyowardojo et al., 2011). We expect that undersampling of males limited our power and, thereby, our sensitivity in identifying smaller differences in physical health risks between autistic and non-autistic males. However, our results support that autistic females and males both have increased risks compared to the non-autistic population and also that risk may vary based on biological sex.

### Strengths

Although previous studies have found increased health risks for autistic individuals, the current research expands the breadth of conditions that are associated with autistic adults. Self-report survey measures tend to attract female respondents disproportionately (Aerny-Perreten et al., 2015; Cheung et al., 2017; Cull et al., 2005; Listyowardojo et al., 2011), allowing us to sample a large group of autistic females. Existing studies of autistic adults use medical, insurance, or census records to determine physical health comorbidities. Thus, their data will include a strong sex bias toward males within their autism population (approximately 3:1 or 4:1). As such, the existing study of physical health of autistic adults fails to consider unique risks of autistic females, due to undersampling of this population; however, prevalence of physical health conditions varies greatly by biological sex in the general population (Oksuzyan et al., 2008; Verbrugge et al., 1987). Our methodology also allowed us to recruit a large, international cohort and, thereby, to reach a greater number of autistic adults across the lifespan, and especially understudied groups: 738 autistic females, an average age of approximately 41 years in both the autistic and control groups, and 588 autistic participants aged 40 years or older. As the largest sample of middle-aged and older autistic females, our results make clear that there are significant health risks to this group, even when taking lifestyle factors into account.

Another advantage of our methodology is that our study had very little missing data; missing data and the biases introduced by imputation are significant issues in retrospective medical record analysis. Model 1 in our study avoided any biases from imputation; while some values for the seven covariates were imputed in Model 2 and Model 3, 2.16% or less of the data for any variable were missing for either the autistic or non-autistic groups.

A final strength of our methodology is that we are the first large-scale study to quantify the effect of lifestyle factors (such as smoking, alcohol use, and BMI) on risk of developing chronic physical health conditions among autistic adults. Specifically, our analyses focus on identifying differences in prevalence of cancers, cardiovascular conditions, respiratory conditions, and diabetic conditions and their relationship to lifestyle factors; collectively, these conditions account for over 70% of worldwide mortality, and our study provides insight into some of the increased physical health risks that may contribute to premature mortality seen among autistic individuals across the spectrum. As our results suggest that these physical health risks remain even after accounting for differences in lifestyle factors, this study may have implications for future healthcare of autistic adults, and the importance of healthcare maintenance checks in preventing premature mortality among autistic individuals.

### Contributing factors

There are several reasons that autistic adults may carry a greater physical health burden than others. As autism is polygenic in nature, there may be overlapping genetic risks from either rare or common variants between autism and physical health conditions. A recent study utilized pathway network analyses to show that genes associated with autism may also provide vulnerability to chronic medical conditions, including cancer, cardiovascular conditions, and metabolic conditions (and specifically Type II diabetes; Wen et al., 2016). We excluded individuals who reported genetic syndromes that are known to have clear physical health risks; however, future research should focus on utilizing both genetic and health data together, to establish relative genetic risk of particular conditions. Another biological factor that may affect risk of developing physical health conditions is dysregulation of sex steroid hormones; autistic individuals may be more likely to have elevated prenatal steroidogenic activity, later hormone dysregulation, and/or hormone-related health conditions in later adulthood (Baron-Cohen et al., 2015, 2019; Berni et al., 2018; Cherskov et al., 2018; Pohl et al., 2014; Ruta et al., 2011; Schwarz et al., 2011), which may in turn increase likelihood of developing obesity and a variety of physical health conditions including cancer, diabetes, and cardiovascular conditions (Berni et al., 2018; Bhupathy et al., 2010; Brand et al., 2011; Cherskov et al., 2018; Mantovani & Fucic, 2014).

Further, while our study attempts to control for some lifestyle factors, these measures are limited in their ability to detail actual lifestyle differences between autistic and non-autistic adults. Our sample only accounts for participants’ currently alcohol consumption frequency and the frequency of their smoking when smoking the most; additionally, we know that the BMI may correspond to lifestyle choices, including diet and exercise, but also may relate to genetic or other unknown factors as well. Future research should focus on establishing lifestyle differences between middle-aged and older autistic and non-autistic adults that could contribute to physical health risks.

Recent research has identified that autistic adults may be at higher risk of negative life experiences (Griffiths et al., 2019) and that, in children, these negative life experiences can affect their health (Rigles, 2017). Finally, differences in the prevalence of physical health conditions may be related to the quality of care received by autistic individuals. There is evidence of increased healthcare utilization among autistic adults, with one study even suggesting that autistic adults may carry double the total annual mean healthcare costs of individuals in the general population (Vohra et al., 2017; Weiss et al., 2018; Zerbo et al., 2018). Despite this, autistic adults report lower satisfaction with patient–provider communication, lower self-efficacy for both general healthcare and chronic conditions, higher odds of unmet healthcare needs related to physical health, and greater odds of using emergency care (Nicolaidis et al., 2013). Further, a recent systematic review found that patient–provider communication, sensory sensitivities, and executive functioning/planning issues served as significant barriers to accessing healthcare for autistic individuals; in addition, negative experiences with healthcare providers and healthcare providers’ lack of knowledge of autism both served as barriers in at least one of the studies included (Mason et al., 2019). Lack of appropriate healthcare services and of useful support may magnify underlying healthcare risks by failing to provide early intervention.

To reduce the risk of physical health comorbidities in autistic adults, future research must focus on establishing the relative contribution of each of these factors to the physical health risks and premature mortality of autistic individuals.

## Conclusion

This study reports evidence of increased physical health risks for autistic adults in the areas of cardiovascular, respiratory, and diabetic conditions. Many of these risks remain for autistic individuals, even when taking alcohol use, smoking, and BMI into account. The 2016 WHO report states that these conditions account for a large proportion of premature mortality in the general population. We know that autistic individuals are at elevated risk of premature mortality. Therefore, future research should focus on further clarifying physical health risks to autistic adults across the lifespan that may contribute to premature mortality, as well as establishing the reasons for increased physical health risks among autistic adults.

## Supplemental Material

sj-jpg-1-aut-10.1177_1362361320953652 – Supplemental material for Increased prevalence of non-communicable physical health conditions among autistic adults Supplemental material, sj-jpg-1-aut-10.1177_1362361320953652 for Increased prevalence of non-communicable physical health conditions among autistic adults by Elizabeth Weir, Carrie Allison, Varun Warrier and Simon Baron-Cohen in Autism

sj-jpg-2-aut-10.1177_1362361320953652 – Supplemental material for Increased prevalence of non-communicable physical health conditions among autistic adults Supplemental material, sj-jpg-2-aut-10.1177_1362361320953652 for Increased prevalence of non-communicable physical health conditions among autistic adults by Elizabeth Weir, Carrie Allison, Varun Warrier and Simon Baron-Cohen in Autism

sj-jpg-3-aut-10.1177_1362361320953652 – Supplemental material for Increased prevalence of non-communicable physical health conditions among autistic adults Supplemental material, sj-jpg-3-aut-10.1177_1362361320953652 for Increased prevalence of non-communicable physical health conditions among autistic adults by Elizabeth Weir, Carrie Allison, Varun Warrier and Simon Baron-Cohen in Autism

sj-jpg-4-aut-10.1177_1362361320953652 – Supplemental material for Increased prevalence of non-communicable physical health conditions among autistic adults Supplemental material, sj-jpg-4-aut-10.1177_1362361320953652 for Increased prevalence of non-communicable physical health conditions among autistic adults by Elizabeth Weir, Carrie Allison, Varun Warrier and Simon Baron-Cohen in Autism

sj-jpg-5-aut-10.1177_1362361320953652 – Supplemental material for Increased prevalence of non-communicable physical health conditions among autistic adults Supplemental material, sj-jpg-5-aut-10.1177_1362361320953652 for Increased prevalence of non-communicable physical health conditions among autistic adults by Elizabeth Weir, Carrie Allison, Varun Warrier and Simon Baron-Cohen in Autism

sj-jpg-6-aut-10.1177_1362361320953652 – Supplemental material for Increased prevalence of non-communicable physical health conditions among autistic adults Supplemental material, sj-jpg-6-aut-10.1177_1362361320953652 for Increased prevalence of non-communicable physical health conditions among autistic adults by Elizabeth Weir, Carrie Allison, Varun Warrier and Simon Baron-Cohen in Autism

sj-jpg-7-aut-10.1177_1362361320953652 – Supplemental material for Increased prevalence of non-communicable physical health conditions among autistic adults Supplemental material, sj-jpg-7-aut-10.1177_1362361320953652 for Increased prevalence of non-communicable physical health conditions among autistic adults by Elizabeth Weir, Carrie Allison, Varun Warrier and Simon Baron-Cohen in Autism

sj-pdf-8-aut-10.1177_1362361320953652 – Supplemental material for Increased prevalence of non-communicable physical health conditions among autistic adults Supplemental material, sj-pdf-8-aut-10.1177_1362361320953652 for Increased prevalence of non-communicable physical health conditions among autistic adults by Elizabeth Weir, Carrie Allison, Varun Warrier and Simon Baron-Cohen in Autism
