# Supplementary figures and images for: Increased prevalence of non-communicable physical health conditions among autistic adults
Source: Autism. 2020 Sep 9;25(3):681–94. doi: 10.1177/1362361320953652 (PMC7610707; doi:10.1177/1362361320953652)

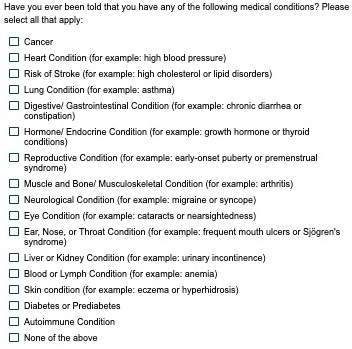

Supplement: sj-jpg-1-aut-10.1177_1362361320953652 – Supplemental material for Increased prevalence of non-communicable physical health conditions among autistic adults [file sj-jpg-1-aut-10.1177_1362361320953652.jpg]

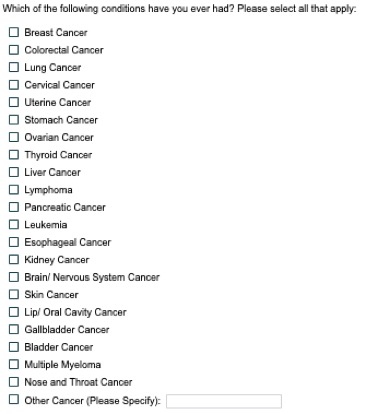

Supplement: sj-jpg-2-aut-10.1177_1362361320953652 – Supplemental material for Increased prevalence of non-communicable physical health conditions among autistic adults [file sj-jpg-2-aut-10.1177_1362361320953652.jpg]

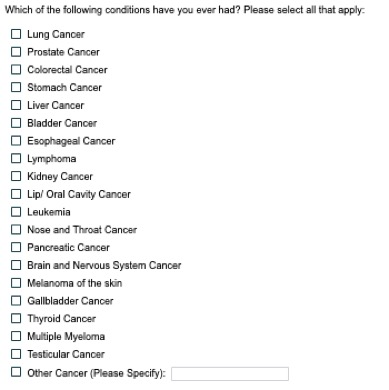

Supplement: sj-jpg-3-aut-10.1177_1362361320953652 – Supplemental material for Increased prevalence of non-communicable physical health conditions among autistic adults [file sj-jpg-3-aut-10.1177_1362361320953652.jpg]

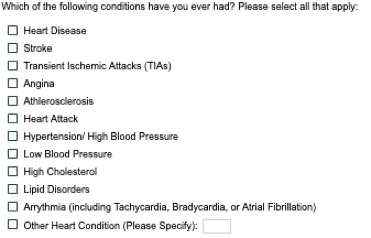

Supplement: sj-jpg-4-aut-10.1177_1362361320953652 – Supplemental material for Increased prevalence of non-communicable physical health conditions among autistic adults [file sj-jpg-4-aut-10.1177_1362361320953652.jpg]

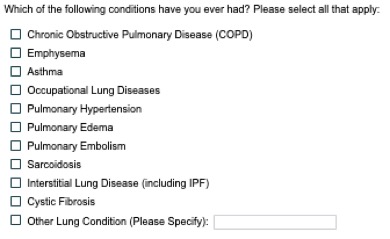

Supplement: sj-jpg-5-aut-10.1177_1362361320953652 – Supplemental material for Increased prevalence of non-communicable physical health conditions among autistic adults [file sj-jpg-5-aut-10.1177_1362361320953652.jpg]

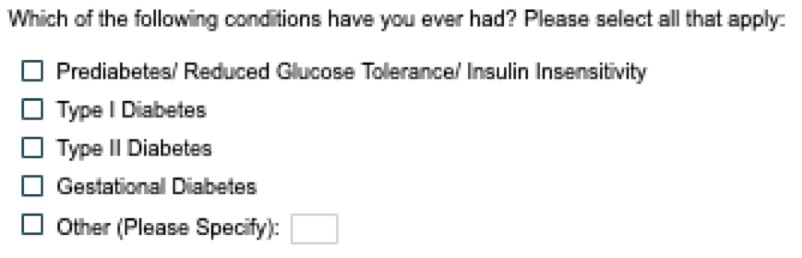

Supplement: sj-jpg-6-aut-10.1177_1362361320953652 – Supplemental material for Increased prevalence of non-communicable physical health conditions among autistic adults [file sj-jpg-6-aut-10.1177_1362361320953652.jpg]

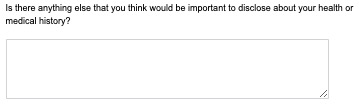

Supplement: sj-jpg-7-aut-10.1177_1362361320953652 – Supplemental material for Increased prevalence of non-communicable physical health conditions among autistic adults [file sj-jpg-7-aut-10.1177_1362361320953652.jpg]
