# Supplementary material for: Increased prevalence of non-communicable physical health conditions among autistic adults
Source: Autism. 2020 Sep 9;25(3):681–94. doi: 10.1177/1362361320953652 (PMC7610707; doi:10.1177/1362361320953652)
Supplement: sj-pdf-8-aut-10.1177_1362361320953652 – Supplemental material for Increased prevalence of non-communicable physical health conditions among autistic adults [file sj-pdf-8-aut-10.1177_1362361320953652.pdf]

Supplementary Table 1: Model 1, Sex-Stratified Health Risks by Condition (Unadjusted)

Conditions	Odds Ratios	95% Confidence Interval	Fisher's Exact Test p-value	False Discovery Rate	Signif. Level
Cancer (Overall)					
Female	0.830	0.517—Inf	0.799	0.882	
Male	0.864	0.484—Inf	0.732	0.801	
Cardiovascular (Overall)					
Female	1.306	1.039—Inf	0.027	0.049	*
Male	1.438	1.082—Inf	0.017	0.049	*
Respiratory (Overall)					
Female	1.898	1.511—Inf	8.821×10^{-7}	4.851×10^{-6}	***
Male	1.067	0.745—Inf	0.417	0.573	
Diabetic (Overall)					
Female	1.450	1.034—Inf	0.035	0.054	▲
Male	1.668	1.015—Inf	0.045	0.098	▲
Low Blood Pressure					
Female	2.941	1.842—Inf	2.135×10^{-5}	5.872×10^{-5}	***
High Blood Pressure					
Female	0.755	0.549—Inf	0.949	0.949	
Male	1.185	0.838—Inf	0.226	0.414	
High Cholesterol					
Female	0.980	0.666—Inf	0.579	0.796	
Male	1.679	1.110—Inf	0.018	0.049	*
Heart Disease					
Male	1.307	0.652—Inf	0.306	0.481	
Arrhythmia					
Female	2.871	1.913—Inf	2.091×10^{-6}	7.667×10^{-6}	***
Male	2.917	1.644—Inf	4.492×10^{-4}	4.942×10^{-3}	**
Asthma					
Female	1.978	1.565—Inf	3.473×10^{-7}	3.821×10^{-6}	***
Male	0.925	0.631—Inf	0.680	0.801	
Type II Diabetes					
Female	0.805	0.455—Inf	0.802	0.882	
Male	3.388	1.374—Inf	8.764×10^{-3}	0.048	*
Prediabetes					
Female	3.153	1.687—Inf	5.405×10^{-4}	1.189×10^{-3}	**
Male	0.767	0.353—Inf	0.801	0.801	

Significance Level: *** ($p < 0.001$), ** ($p < 0.01$), * ($p < 0.05$), ▲ ($p < 0.10$)

Supplementary Table 2: Model 2, Sex-Stratified Health Risks by Condition (Adjusting for Age, Ethnicity, and Education-Level)

Conditions	Odds Ratios	95% Confidence Interval	Binomial Logistic Regression p-value	False Discovery Rate	Signif. Level
Cancer (Overall)					
Female	1.098	0.627—1.911	0.741	0.815	
Male	0.840	0.431—1.628	0.603	0.830	
Cardiovascular (Overall)					
Female	1.511	1.139—2.010	4.192×10^{-3}	6.587×10^{-3}	**
Male	1.590	1.104—2.302	0.013	0.069	▲
Respiratory (Overall)					
Female	2.016	1.541—2.647	2.636×10^{-7}	1.450×10^{-6}	***
Male	0.955	0.623—1.464	0.832	0.866	
Diabetic (Overall)					
Female	1.835	1.223—2.771	3.285×10^{-3}	6.022×10^{-3}	**
Male	1.731	0.979—3.132	0.059	0.163	
Low Blood Pressure					
Female	2.900	1.716—5.073	5.046×10^{-5}	1.110×10^{-4}	***
High Blood Pressure					
Female	0.965	0.650—1.426	0.857	0.857	
Male	1.292	0.850—1.975	0.230	0.423	
High Cholesterol					
Female	1.435	0.892—2.313	0.136	0.188	
Male	1.523	0.924—2.542	0.099	0.218	
Heart Disease					
Male	1.146	0.520—2.593	0.736	0.866	
Arrhythmia					
Female	2.941	1.854—4.783	2.855×10^{-6}	1.047×10^{-5}	***
Male	2.979	1.556—6.047	7.885×10^{-4}	8.674×10^{-3}	*
Asthma					
Female	2.069	1.571—2.736	1.850×10^{-7}	1.450×10^{-6}	***
Male	0.832	0.527—1.313	0.430	0.676	
Type II Diabetes					
Female	1.237	0.637—2.386	0.526	0.643	
Male	2.483	0.995—7.150	0.051	0.163	
Prediabetes					
Female	4.337	2.147—9.395	2.523×10^{-5}	6.937×10^{-5}	***
Male	1.075	0.461—2.483	0.866	0.866	

Significance Level: *** ($p < 0.001$), ** ($p < 0.01$), * ($p < 0.05$), ▲ ($p < 0.10$)

Supplementary Table 3: Model 3, Sex-Stratified Health Risks by Condition (Adjusting for Age, Ethnicity, Education-Level, BMI, Alcohol, and Smoking)

Conditions	Odds Ratios	95% Confidence Interval	Binomial Logistic Regression p-value	False Discovery Rate	Signif. Level
Cancer (Overall)					
Female	1.068	0.605—1.872	0.820	0.902	
Male	0.739	0.367—1.473	0.390	0.612	
Cardiovascular (Overall)					
Female	1.383	1.033—1.854	0.029	0.046	*
Male	1.542	1.056—2.262	0.025	0.136	
Respiratory (Overall)					
Female	2.054	1.557—2.719	2.830×10^{-7}	1.557×10^{-6}	***
Male	0.903	0.583—1.399	0.647	0.792	
Diabetic (Overall)					
Female	1.692	1.109—2.599	0.015	0.027	*
Male	1.509	0.822—2.830	0.186	0.511	
Low Blood Pressure					
Female	2.541	1.494—4.467	4.821×10^{-4}	1.061×10^{-3}	**
High Blood Pressure					
Female	0.873	0.578—1.313	0.514	0.628	
Male	1.158	0.743—1.812	0.517	0.711	
High Cholesterol					
Female	1.382	0.850—2.248	0.192	0.263	
Male	1.451	0.867—2.456	0.157	0.511	
Heart Disease					
Male	1.121	0.501—2.562	0.782	0.860	
Arrhythmia					
Female	2.928	1.829—4.804	5.002×10^{-6}	1.834×10^{-5}	***
Male	3.085	1.561—6.458	9.726×10^{-4}	0.011	*
Asthma					
Female	2.092	1.576—2.789	2.554×10^{-7}	1.557×10^{-6}	***
Male	0.796	0.499—1.267	0.335	0.612	
Type II Diabetes					
Female	1.028	0.510—2.055	0.938	0.938	
Male	1.799	0.688—5.266	0.237	0.522	
Prediabetes					
Female	4.379	2.102—9.772	5.011×10^{-5}	1.378×10^{-4}	***
Male	1.020	0.419—2.474	0.964	0.964	

Significance Level: *** ($p < 0.001$), ** ($p < 0.01$), * ($p < 0.05$), ▲ ($p < 0.10$)

Supplementary Table 4: Interaction of Age and Diagnosis in Autistic and Non-Autistic Females and Males, respectively

Conditions	Age by Diagnosis Interaction Coefficient	p-value	False Discovery Rate	Age Coefficient	p-value	False Discovery Rate
Cancer (Overall)						
Female	-8.395×10^{-3}	0.672	0.878	0.066	4.185×10^{-8}	1.151×10^{-7}
Male	-0.028	0.187	0.687	0.063	1.038×10^{-5}	3.805×10^{-5}
Cardiovascular (Overall)						
Female	-0.023	0.021	0.228	0.056	1.110×10^{-16}	6.106×10^{-16}
Male	-4.459×10^{-4}	0.971	0.971	0.057	1.091×10^{-10}	1.200×10^{-9}
Respiratory (Overall)						
Female	-1.453×10^{-3}	0.878	0.878	-1.511×10^{-3}	0.824	0.824
Male	4.317×10^{-3}	0.749	0.971	3.496×10^{-3}	0.730	0.775
Diabetic (Overall)						
Female	0.013	0.361	0.878	0.042	1.173×10^{-5}	2.580×10^{-5}
Male	4.356×10^{-3}	0.823	0.971	0.029	0.056	0.084
Low Blood Pressure						
Female	0.025	0.178	0.654	4.622×10^{-3}	0.754	0.824
High Blood Pressure						
Female	-9.833×10^{-3}	0.490	0.878	0.071	0	0
Male	-0.012	0.417	0.971	0.053	2.012×10^{-7}	1.107×10^{-6}
High Cholesterol						
Female	5.345×10^{-3}	0.762	0.878	0.070	6.404×10^{-11}	2.348×10^{-10}
Male	0.039	0.033	0.205	0.039	2.477×10^{-3}	6.812×10^{-3}
Heart Disease						
Male	0.068	0.037	0.205	0.050	5.396×10^{-3}	0.012
Arrhythmia						
Female	-2.667×10^{-3}	0.867	0.878	0.020	0.123	0.181
Male	9.363×10^{-3}	0.667	0.971	0.035	0.054	0.084
Asthma						
Female	3.451×10^{-3}	0.724	0.878	-9.248×10^{-3}	0.194	0.237
Male	5.660×10^{-4}	0.969	0.971	-3.064×10^{-3}	0.775	0.775
Type II Diabetes						
Female	0.013	0.596	0.878	0.061	1.656×10^{-5}	3.035×10^{-5}
Male	0.039	0.757	0.971	0.027	0.169	0.206
Prediabetes						
Female	-0.010	0.106	0.582	0.043	0.131	0.181
Male	-6.969×10^{-3}	0.807	0.971	0.037	0.061	0.084

Significance Level: *** ($p < 0.001$), ** ($p < 0.01$), * ($p < 0.05$), ▲ ($p < 0.10$)

These are the full results for our analysis on the interaction between age and diagnosis. Although there were marginally significant interactions between age and diagnosis for cardiovascular conditions overall for autistic females (compared to non-autistic females), as well as for high

cholesterol and heart disease for autistic males (compared to non-autistic males), these results did not survive correction.
